# On implementation of DCTCP on three-tier and fat-tree data center network topologies

**DOI:** 10.1186/s40064-016-2454-4

**Published:** 2016-06-17

**Authors:** Saima Zafar, Abeer Bashir, Shafique Ahmad Chaudhry

**Affiliations:** Department of Electrical Engineering, National University of Computer and Emerging Sciences, FAST-NU, Block B, Faisal Town, Lahore, Pakistan; Department of Computer Science, Dhofar University, Salalah, Oman

**Keywords:** Data center network, Three-tier topology, Fat tree topology, Transport layer protocol

## Abstract

A data center is a facility for housing computational and storage systems interconnected through a communication network called data center network (DCN). Due to a tremendous growth in the computational power, storage capacity and the number of inter-connected servers, the DCN faces challenges concerning efficiency, reliability and scalability. Although transmission control protocol (TCP) is a time-tested transport protocol in the Internet, DCN challenges such as inadequate buffer space in switches and bandwidth limitations have prompted the researchers to propose techniques to improve TCP performance or design new transport protocols for DCN. Data center TCP (DCTCP) emerge as one of the most promising solutions in this domain which employs the explicit congestion notification feature of TCP to enhance the TCP congestion control algorithm. While DCTCP has been analyzed for two-tier tree-based DCN topology for traffic between servers in the same rack which is common in cloud applications, it remains oblivious to the traffic patterns common in university and private enterprise networks which traverse the complete network interconnect spanning upper tier layers. We also recognize that DCTCP performance cannot remain unaffected by the underlying DCN architecture hence there is a need to test and compare DCTCP performance when implemented over diverse DCN architectures. Some of the most notable DCN architectures are the legacy three-tier, fat-tree, BCube, DCell, VL2, and CamCube. In this research, we simulate the two switch-centric DCN architectures; the widely deployed legacy three-tier architecture and the promising fat-tree architecture using network simulator and analyze the performance of DCTCP in terms of throughput and delay for realistic traffic patterns. We also examine how DCTCP prevents incast and outcast congestion when realistic DCN traffic patterns are employed in above mentioned topologies. Our results show that the underlying DCN architecture significantly impacts DCTCP performance. We find that DCTCP gives optimal performance in fat-tree topology and is most suitable for large networks.

## Introduction

A data center is a facility that houses computational and storage systems which are interconnected through a communication network. Although the earlier data centers comprised of interconnected servers and related equipment, the hierarchical design of these servers to match the ever increasing size of facilities has lately been termed as the Internet data center or “data center”. In addition to computational and storage systems, it includes power supply equipment, communication network, air conditioning, security systems and other related devices and can span over an area as large as a small town. These data centers serve a large number of popular services in Internet such as search engines (e.g. Google), Internet commerce (e.g. Amazon and e-Bay), web based e-mail (e.g. yahoo mail), social networking (e.g. Myspace and Facebook) and video sharing (e.g. YouTube). They can also serve other types of network services that demand heavy infrastructure which requires the service providers to procure, establish and maintain server farms. The Data Center Network (DCN) lies at the core of a data center as it connects a large number of servers at various hierarchies through switches. In large data centers, the DCN can connect hundreds or thousands of servers to support various applications and cloud computing, requiring highly efficient and scalable design. Over the last decade, research efforts were directed to design novel DCN architectures or network models to increase efficiency and support scalability. Some of the notable DCN architectures are the legacy three-tier, fat-tree, DCell, BCube, CamCube, FiConn and Jelly fish (Al-Fares et al. 2008; Greenberg et al. 2009b; Guo et al. 2009; Bilal et al. 2012; Bilal et al. 2013; Liu et al. 2013; Greenberg et al. 2008).

Due to tremendous growth and ubiquitous demands of data centers, DCN faces challenges such as inadequate buffer space in switches, bandwidth limitations, congestion and security issues etc. For reliable data communication and congestion control, TCP is an important transport layer protocol because it is a time-tested Internet protocol. However due to special demands and requirements of DCN, TCP is deemed unsuitable for these networks. Data center network traffic comprises two types of flows with conflicting service requirements. The long flows require high throughput and short flows require low latency. TCP does not cater these conflicting requirements of data center traffic and hence research efforts are underway for suggesting improvements in TCP or designing new DCN transport protocols (Al-Fares et al. 2010; Chen et al. 2012; Prakash et al. 2012; Vasudevan et al. 2009; Wu et al. 2013; Raiciu et al. 2011; Foued et al. 2007; Alizadeh et al. 2010). DCTCP is a transport protocol especially designed for DCN which aims at reducing latency and increasing throughput and burst tolerance by employing Active Queue Management (AQM) such that packets experiencing queuing delay longer than a certain threshold are marked with explicit congestion notification (Alizadeh et al. 2010).

Although DCTCP has been tested over two-tier tree-based DCN topology for traffic pattern restricted to servers in the same rack which is a common traffic pattern in cloud applications, in university and private enterprise networks 40–90 % traffic leaves the rack and traverses the network’s interconnect (Benson et al. 2009). Moreover, issues like TCP outcast occurs due to severe unfairness attributable to multi-rooted tree topologies in data center networks (Prakash et al. 2012). These facts necessitate analysis of DCTCP over complete unabridged DCN architecture including upper layer switches and links and traffic pattern spanning complete network. We also observe that there is a need to test and compare DCTCP performance when implemented across different DCN architectures. In this research, we simulate unabridged topologies of the two switch-centric DCN architectures; the legacy three-tier topology and the more promising fat-tree topology using network simulator (NS2) and analyze the performance of TCP and DCTCP in terms of throughput and delay for different traffic patterns. We find that although DCTCP outperforms TCP in both DCN architectures, the underlying DCN architecture considerably affects DCTCP performance. When network size is small DCTCP performance is almost similar in both topologies but as DCN scales to a large network size, DCTCP performs exceptionally well in fat-tree architecture in terms of both throughput and delay. We conclude that DCTCP performance is largely affected by the changes in DCN architecture therefore DCN architecture should be an important design consideration when proposing solutions for congestion and related issues in data center networks.

Our major contributions include: (a) simulating unabridged three-tier and fat-tree DCN architectures; (b) analyzing DCTCP for within-rack and out-of-rack traffic patterns; and (c) analyzing DCTCP for incast and outcast congestion for out-of-rack traffic pattern over three-tier and fat-tree topologies. The rest of the paper is organized as follows. Second section explains the background for our research and an overview of the DCTCP protocol. Third section presents the results of our analysis and fourth section presents the discussion. In fifth section we discuss the related work and finally, sixth section concludes the paper.

## Background

In this section, we explain the three-tier and fat-tree DCN architectures indicating the merits of fat-tree topology. We discuss traffic characteristics of data center networks and indicate the different traffic patterns commonly found in diverse data center applications. We explain the incast and outcast congestion in data center networks and discuss basic features of DCTCP.

### Data center network architectures

Data center network architectures are typically classified into two categories: switch-centric and server-centric. In switch-centric DCNs, the routing intelligence is placed on switches and each server connects to the network through a single port. In server-centric DCNs, the routing intelligence is placed on servers which connect to the network through multiple ports while switches serve merely as cross-bars. Although a number of architectures in both categories have been proposed in order to achieve scalability, efficiency, reliability, cost minimization etc. in addition to some dual-centric architectures combining the best of both categories, the legacy three-tier tree-based architecture continues to be the most widely deployed and fat-tree being the most promising in terms of scalability, robustness and cost (Bilal et al. 2013). Both of these architectures are switch-centric. A typical data center network consists of access layer, aggregation layer and core layer. It consists of routers and switches, in two-level or three-level hierarchy. In two-level hierarchy there are no aggregation switches while three-level hierarchy includes all three layers. The most realistic and practical DCN simulation involves three-level architectures. We briefly explain the legacy three-tier and fat-tree architectures below:

#### Three-tier data center network architecture

**Fig. 1:**
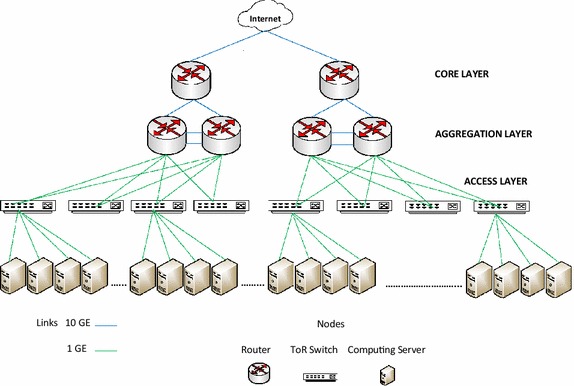
Three-tier data center network topology

The three-tier design of data center network comprises of tree or hierarchy of switches or routers. The root of the tree forms the *core layer*, the middle tier forms the *aggregation layer*, and the leaves of the tree form the *edge/access layer*. The switches at the access layer have some 1 GigE ports (typically 48-288) and some 10 GigE ports for uplink connectivity with the switches of the aggregation layer. The upper layer switches have 10 GigE ports (typically 32-128) and reasonable capacity. Figure 1 shows a section of three-tier DCN topology with switches at different layers and 1 GigE links connecting servers to access layer switches and 10 GigE links connecting switches and routers of upper layers. The switches in access layer are low cost Top Of Rack (TOR) switches which are Ethernet switches connecting servers in the same rack (typically 20–40 servers) through 1 GigE links. These access layer switches are connected to the aggregation layer switches through 10 GigE links. The aggregation layer switches are connected to the core layer switches. The core layer has core layer switches and one or more border routers providing connectivity between data center network and Internet. Normally the aggregation layer has a load balancer.

Some of the drawbacks of this design are oversubscription less than 1:1 due to prohibitive costs. An oversubscription of 1:1 means that all servers communicate with other arbitrary servers at full bandwidth of their network interface. Typical oversubscription in this topology is 2.5:1 or 8:1 (Al-Fares et al. 2008). Large data centers have multi-rooted core switches with multiple core switches which requires multipath routing techniques. This leads to oversubscription, limiting multiplicity of paths, and excessively large routing table entries increasing lookup latency. The most serious shortcoming of this topology is excessive cost due to 10 GigE switches in the upper layers. These shortcomings were identified and resolved by Al-Fares et al. (2008) who proposed fat-tree topology discussed below.

#### Fat-tree data center network architecture

**Fig. 2:**
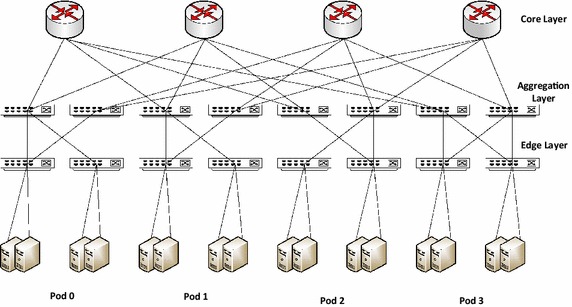
Fat-tree data center network topology

The fat-tree data center network design incorporates the low cost Ethernet commodity switches to form a *k*-ary fat-tree (Al-Fares et al. 2008). As shown in Fig. 2, there are *k* pods, each having 2 layers of *k*/2 switches. Each switch in the lower layer is *k*-port connecting directly to *k*/2 servers through *k*/2 ports and connecting with *k*/2 ports of aggregation layer through remaining *k*/2 ports. There are (*k*/2)^2^*k*-port core switches with one port connecting to each pod. Generally, a fat-tree with *k*-port switches supports *k*^3^/4 servers. The fat-tree topology supports the use of identical, commodity switches in all layers offering multiple-times cost reduction as compared to tier architectures. This design employs two-level route lookups to assist multi-path routing. In order to prevent congestion at a single port due to concentration of traffic to a subnet and to keep the number of prefixes to a limited number, two-level routing tables are used that spread outgoing traffic from a pod evenly among core switches by using the low-order bits of the destination IP address.

### Traffic characteristics of data center networks

In this subsection, we summarize the traffic characteristics of data center networks which we studied in order to understand realistic and common DCN traffic patterns for our simulations. Investigation of data center traffic characteristics is normally done by analyzing the Simple Network Management Protocol (SNMP) data from production data centers and examining the temporal and spatial patterns of traffic volumes and loss rates in switches. Benson et al. (2009, 2010) conducted this study by analyzing the SNMP data from 19 corporate and enterprise data centers. They studied link utilization and packet loss in the core, aggregation and edge layer switches. They report that roughly 60 % of the core and edge links are actively being used with utilization in core significantly higher than the lower layers mainly due to a smaller number of core links multiplexing traffic from lower layers. An important observation is low losses at the core layer despite higher utilization which can be attributed to the use of 10 GigE links and ports at the core layer in three-tier topology. In fat-tree topology, due to same capacities of all switches, core switches are expected to experience higher losses. Another observation is that a small fraction of links experience higher losses than other links. They suggest splitting traffic uniformly across all links in order to avoid under-utilized and over-utilized links. They observe the On–Off traffic pattern at the edge switches and also indicate the presence of burst losses. Kandula et al. (2009) analyzed the nature of data center network traffic and studied traffic patterns, congestion, flow characteristics and TCP Incast. They observe that the median numbers of correspondents for a server are two other servers within its rack and four servers outside the rack. Ersoz et al. (2007) studied network traffic in a cluster-based multi-tier data center which according to them is the most cost effective scheme to design data center networks.

Overall data center network traffic is classified as:

1. Inter-data center traffic: This traffic is between two data center networks. It is studied in detail by Chen et al. (2011) who used network traces gathered at five major Yahoo! data centers. This type of traffic is not the focus of our work.
2. Intra-data center traffic: This is the traffic within a single data center network. Traffic is basically the flow of packets or data. These flows can be one-to-one, one-to-many, many-to-one or many-to-many. Also in DCN these flows can be classified as long-duration flows with large number of packets called elephant or long flows and short-duration flows with small number of packets called mice or short flows. Elephant flows require high throughput while mice flows are short control flows demanding low delay.

Benson et al. (2010) analyzed SNMP statistics from ten 2-tier and 3-tier data centers belonging to three different types of organizations and summarized their findings which are as follows: Majority of flows in data centers are small-sized lasting for less than few hundreds of milliseconds. In cloud data centers, 75 % of traffic remains within a rack while in university and private enterprise data centers, 40–90 % of traffic leaves the rack passing through the network. Losses are more at the aggregation layer than other layers. An important observation is that utilizations within the core and aggregation layers are higher (all links with losses having <30 % average utilization) than at the edge layer which has lower utilization (links with losses having 60 % utilization). This shows that losses in upper layers cannot be ignored and there is a need to analyze out-of-rack traffic patterns which we do not see in DCTCP analysis in Alizadeh et al. (2010). We test DCTCP for both within-rack and out-of-rack traffic patterns.

### TCP incast and TCP outcast

**Fig. 3:**
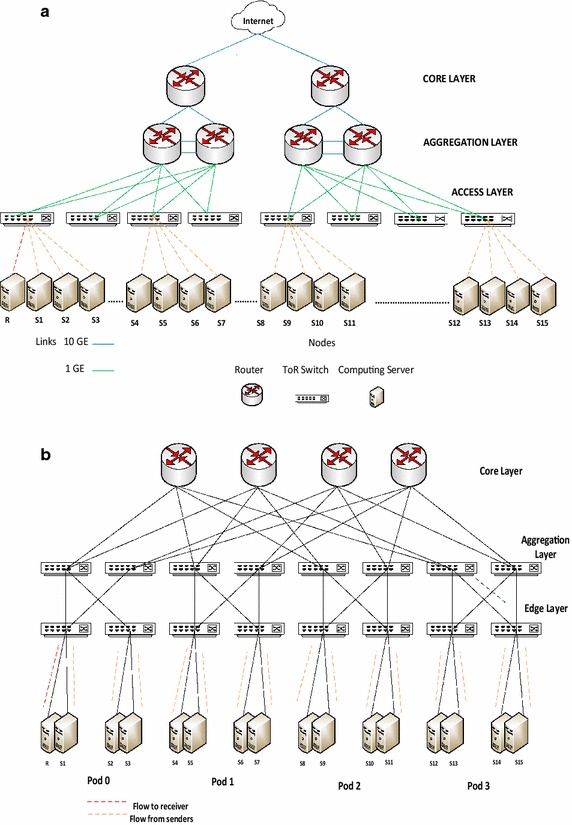
Incast congestion in data center network in **a** three-tier topology and **b** fat-tree topology

TCP incast and TCP outcast congestion results due to two different scenarios of data center traffic which severely degrade throughput and are discussed in detail in Chen et al. (2012) and Prakash et al. (2012) along with their solutions. We simulate and analyze both incast and outcast for three-tier and fat-tree topologies of DCN with traffic patterns spanning upper layers. In this subsection, we briefly discuss the incast and outcast scenarios. TCP incast is a condition which affects network throughput in many-to-one traffic pattern which is quite common in data center networks. This condition occurs when multiple senders send data to the same receiver as shown in Fig. 3. The link between the switch and the receiver becomes the bottleneck resulting in throughput far below the link bandwidth (Chen et al. 2012). Keeping in view the DCN topology, this condition occurs at the TOR switch to which the receiver is connected. Incast congestion can develop both due to within-rack traffic which means all senders are connected to the same TOR switch to which the receiver is connected as well as due to out of rack traffic when senders are spread out in the network but they all send data to the same receiver. The incast problem due to these two scenarios can have different impact on network throughput/delay and needs separate analysis in both three-tier and fat-tree topologies. Figure 3 shows the scenario when senders are scattered in the network connected to different TOR switches for both topologies.

**Fig. 4:**
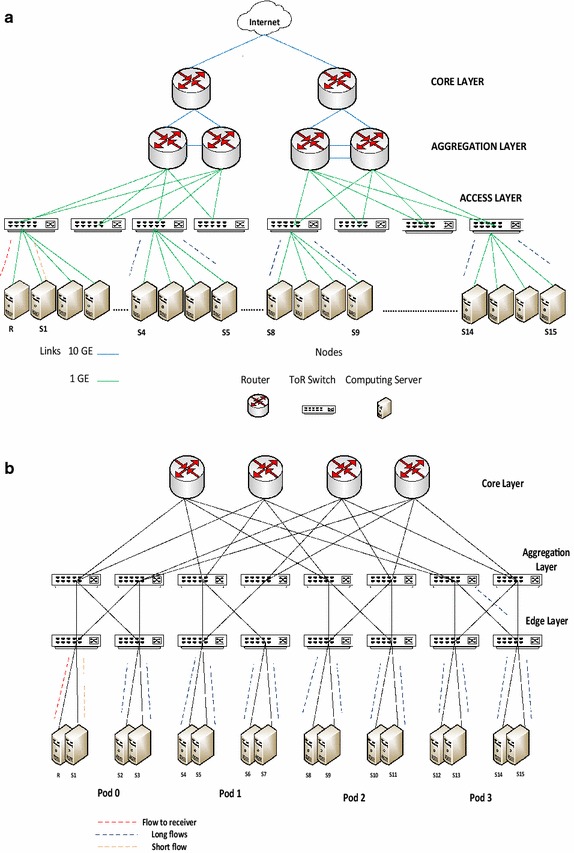
Outcast congestion in data center network in **a** three-tier topology and **b** fat-tree topology

TCP outcast is a frequent problem which occurs in a multi-rooted tree topology when several large and small TCP flows turn up at several input ports of a switch and these flows contest for the same output port (Prakash et al. 2012). This scenario is shown in Fig. 4. In this scenario, large TCP flows are queued at the output port whereas the remaining small TCP flows are dropped. This problem occurs because the commodity switches used in networks use tail-drop queue management scheme which exhibit a phenomenon called port blackout where a series of packets from one port are dropped. This results in severe unfairness in network resource sharing. Similar to incast scenario, outcast congestion also requires separate analysis for senders connected to the same TOR switch and for senders connected to different TOR switches. The second traffic pattern is shown in Fig. 4 for both topologies.

### DCTCP

In this subsection, we briefly explain Data Center TCP (DCTCP) (Alizadeh et al. 2010, 2011). DCTCP is a transport layer protocol especially designed for data centre networks. As discussed earlier, the DCN applications have two types of flows: large flows necessitating high throughput and small flows necessitating low delay. The data traffic inside DCN is classified as query (2 KB-20 KB), short messages (50 KB – 1 MB) and large flows (1 MB – 50 MB). Most of the flows in data centers are small (less than or equal to 10 KB). These requirements are not handled efficiently by the conventional TCP. DCTCP solves this issue in order to meet the requirements of DCN applications. DCTCP achieves low latency, high throughput and high burst tolerance. DCTCP reacts according to the level of congestion and reduces the window size based on fraction of the marked packets. It uses an Active Queue Management (AQM) policy in which the router marks the packet with Explicit Congestion Notification (ECN) rather than dropping it when the number of packets that are queued exceeds the marking threshold (*K*). ECN is used by DCTCP for the early detection of congestion instead of waiting for segment loss to occur. DCTCP algorithm has three main components discussed below:

#### Marking at the switch

DCTCP uses a simple active queue management scheme in which the switch detects the congestion and sets the Congestion Encountered (CE) codepoint in the IP header. If queue occupancy is greater than a marking threshold (*K*) upon the arrival of an incoming packet, it is marked with CE codepoint. This allows the sender to know about the queue overshoot.

#### ECN-echo at the receiver

The receiver echoes the congestion information back to the sender using the ECN-Echo (ECE) flag of TCP header. ECN-Echo (ECE) flag is set by the receiver in the series of ACKs until it receives confirmation from the sender. The receiver tries to convey back to the sender exact sequence of marked packets.

#### Controller at the sender

The sender reacts to the congestion indication by (ECE) flag in the received ACKs from the receiver by reducing the TCP congestion window (*cwnd*). The estimate of fraction of packets marked is represented by *α* and is updated once per Round Trip Time (*RTT)* as:

1 $$\alpha \leftarrow \left( {1 - g} \right) \times \alpha + g \times F$$

where *F* is the fraction of packets marked in the last window of data, *g* is the weight given to new samples against the past in the estimation of *α* and it is 0 < *g* < 1. From Eq. (1) we get to know that through *α*, we can estimate the probability that the queue size is greater than the threshold (*K*). If *α* is close to 0 it means low level of congestion and if close to 1 it indicates high level of congestion.

The conventional TCP cuts its window size by a factor of 2 in response to a marked ACK but DCTCP uses *α* to reduce its window size as mentioned below:

2 $$cwnd \leftarrow cwnd \times \left( {1 - \alpha /2} \right)$$

when *α* is near 0 it means low level of congestion and the congestion window is only slightly reduced but when there is high level of congestion (*α* is near 1), DCTCP cuts its window size by half just like TCP.

## Results

This section is divided into three parts. First, we explain our characterization of the three-tier and fat-tree DCN topologies in Network Simulator (NS2). Second, using realistic DCN traffic patterns, we evaluate DCTCP performance in terms of throughput and delay over the two DCN topologies. Finally we evaluate DCTCP performance for TCP incast and TCP outcast problems.

### Characterizing three-tier and fat-tree DCN topologies

We simulate the topologies discussed in “Data center network architectures” section. The three-tier DCN topology that we simulate comprises of the core layer, aggregation layer and edge layer such that 48-port 1 GigE TOR switches and 128-port 10 GigE aggregation and core layer switches are used. Total number of TOR servers is 4096. The links between servers and TOR switches are 1 Gbps Ethernet links while all upper links are 10 Gbps Ethernet. The fat-tree DCN topology is simulated such that 48-port 1 GigE switches and 1 Gbps Ethernet links are used at all layers.

The simulation parameters are listed in Table 1.

**Table 1:** Parameters for simulation of three-tier and fat-tree DCN topologies

Parameter	Value
Number of nodes	16–4096
Number of pods	4–72
Packet size	1024 bytes
Simulation time	10–1000 s approx.
Communication pair selection	Random selection with uniform probability
Flow pattern of traffic for “DCTCP on three-tier and fat-tree topologies” section	Exponential random traffic
Buffer size	4 MB for 1 GigE and 8 MB for 10 GigE switches
Threshold for queue size (*K* for DCTCP)	65

Some of the simulation details are discussed below:

#### Routing protocol

In three-tier DCN topology simulation, Global Routing Protocol with Equal Cost Multi Path (ECMP) is used. Global Routing Protocol is used when ECMP has to be used in simulation. In this type of routing protocol, flows heading to the same destination can split paths between two equal cost paths. This protocol works like load balancer in the network which efficiently distributes the load and ultimately provides high throughput. In fat-tree DCN topology simulation, two-level routing protocol, Nix Vector Routing Protocol is used to handle different types of situations (Bilal et al. 2012; Nix-Vector routing 2012). In Nix Vector Routing Protocol, routing path is included in the packet header. In the very first transmission, sender sets the path and all other packets have that entire set path included in their headers. This type of routing is used in large-scale network topologies. This protocol provides improved performance in terms of memory usage and simulation run time when dealing with a large number of nodes.

#### Throughput and delay calculation

The formulas used to compute average network throughput and average packet delay are given below (Bilal et al. 2012):

#### Average network throughput

Average network throughput is calculated using the given formula:

3 $$\tau = \frac{{\left( {\mathop \sum \nolimits_{i = 1}^{n} \left( {P_{i} } \right) \times S} \right)}}{{D_{agg} }}$$

where $\tau$ is average network throughput, *P*_*i*_ is *ith* received packet, *S* is packet size in bits and *D*_*agg*_ is the aggregate packets delay.

#### Average packet delay

Average packet delay in the network is calculated using the formula:

4 $$D_{avg} = \frac{{D_{agg} }}{n}$$

5 $$D_{agg} = \mathop \sum \limits_{j = 1}^{n} d^{j}$$

*D*_*agg*_ is the aggregate delay of all received packets, *d*_*j*_ is the delay of *jth* packet and *n* is the total number of packets received in the network.

### DCTCP on three-tier and fat-tree topologies

**Fig. 5:**
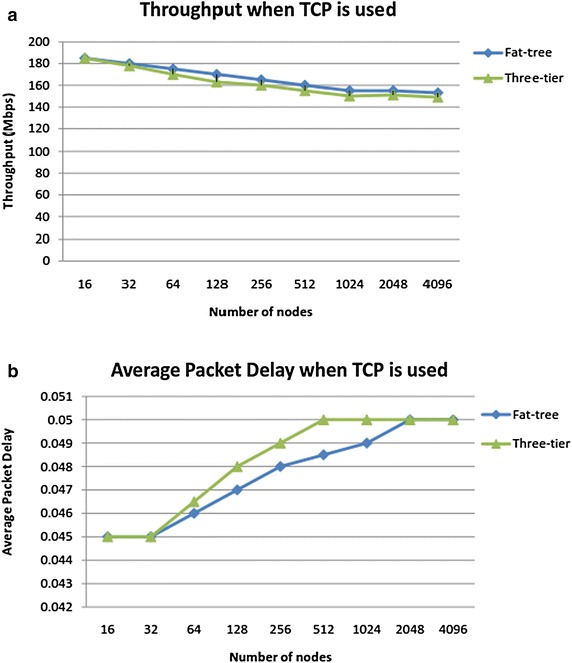
**a** TCP throughput and **b** TCP delay in three-tier and fat-tree topologies

**Fig. 6:**
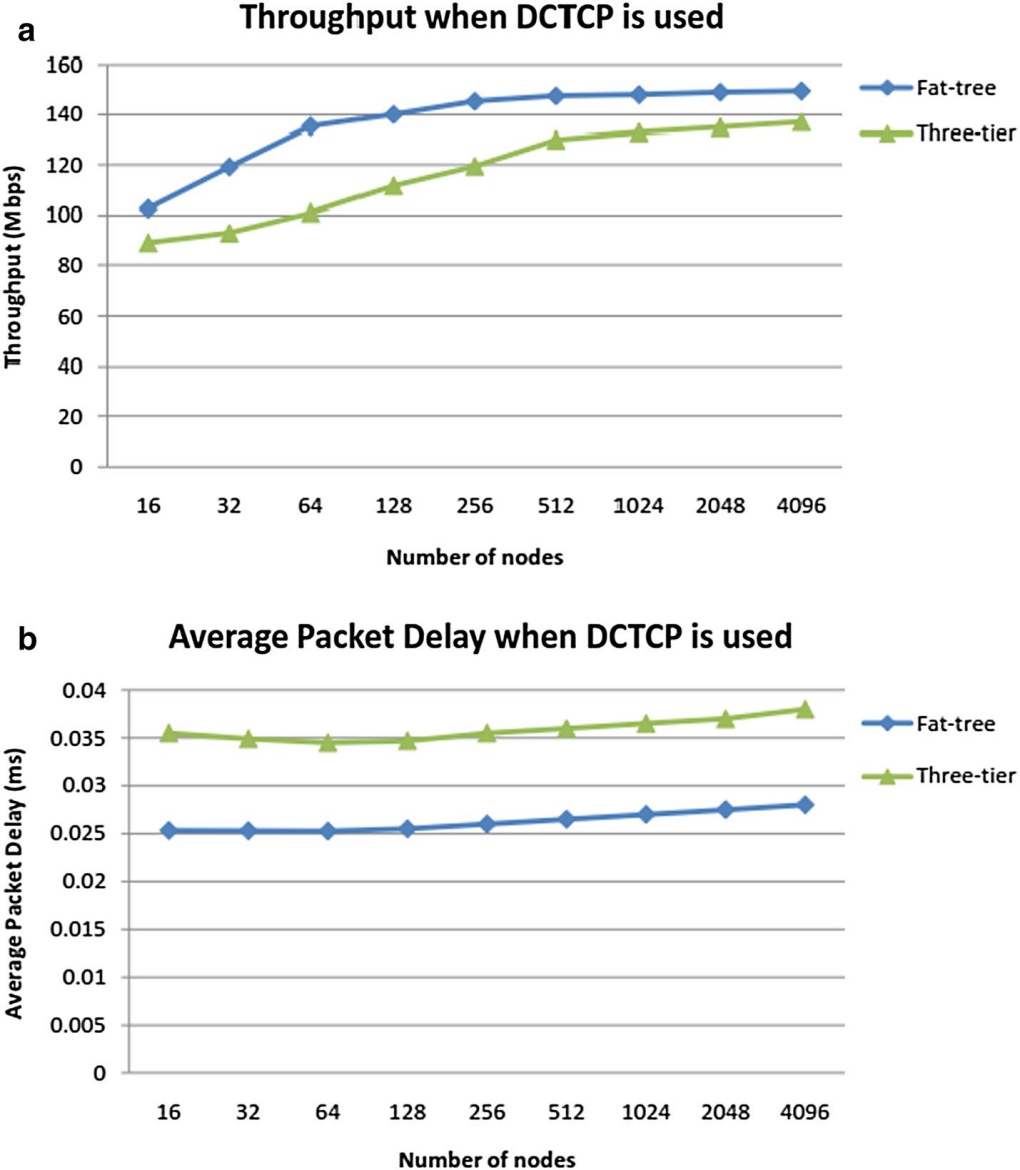
**a** DCTCP throughput and **b** DCTCP delay in three-tier and fat-tree topologies

After simulating the three-tier and fat-tree DCN topologies as discussed above, our first experiment is to generate DCN traffic comprising of mixed flows from arbitrary senders to arbitrary receivers and observe throughput and delay when TCP is used as transport protocol at all network nodes. We use mixed traffic patterns including one-to-one, and one-to-many, many-to-one and many-to-many. The throughput is shown in Fig. 5a for both topologies. We repeat this experiment with DCTCP as the transport protocol and the results are shown in Fig. 6. These results are explained in the “Discussion” section.

### Incast and outcast congestion

**Fig. 7:**
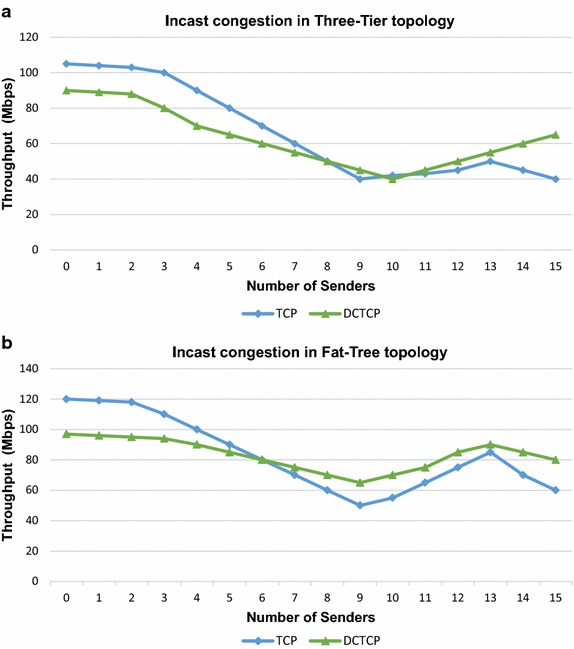
Analysis for incast congestion in **a** three-tier topology and **b** fat-tree topology

**Fig. 8:**
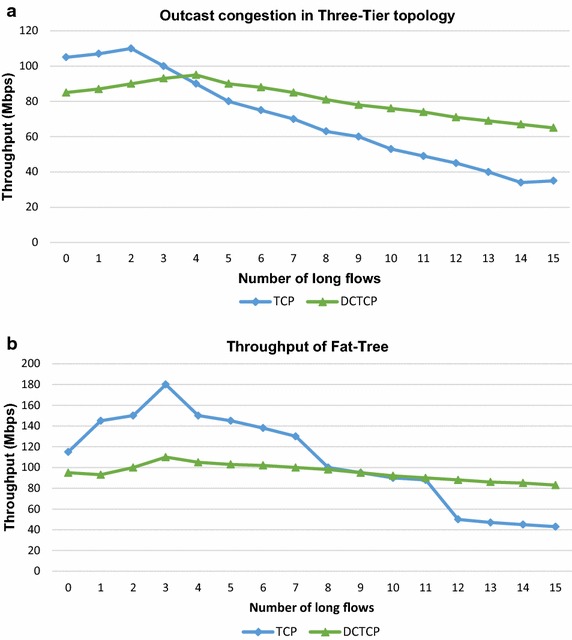
Analysis for outcast congestion in **a** three-tier topology and **b** fat-tree topology

TCP incast congestion can develop at a TOR switch for many-to-one traffic pattern when multiple users send data to a single receiver connected to the switch. The bottleneck is the link between the TOR switch and the receiver. Incast congestion for within rack traffic has been studied in Alizadeh et al. (2010). In this experiment, we simulate incast scenario by generating many-to-one traffic from multiple senders scattered in the network to a single receiver. Figure 7 shows the results of this experiment. We also simulate outcast scenario for out-of-rack traffic as shown in Fig. 4 and observe the impact in both topologies. Figure 8 shows the results of our experiment. These results are explicated in the “Discussion” section.

## Discussion

Average throughput for TCP decreases as the number of flows increase because we notice congestion buildup early on especially in three-tier topology. As shown in Fig. 5a, throughput degradation is relatively less in fat-tree topology because it offers redundant links/switches which ease the pressure. Figure 5b shows the average delay experienced by packets when TCP is implemented in both topologies and we observe that average delay increases almost linearly as queue builds up and it is also worst for three-tier topology than fat-tree. Figure 6a shows that when DCTCP is implemented, the network throughput is high in both topologies due to late queue buildup in switch buffers. The throughput tends to flatten only after a large number of flows. Similarly as shown in Fig. 6b, the average packet delay is reasonably smooth when DCTCP is used. DCTCP performance is markedly superior in fat-tree topology than the three tier topology. Thus DCTCP gives noticeably good performance when used in fat-tree topology because fat-tree employs commodity switches at all layers and DCTCP efficiently uses switches and links to alleviate congestion. Also, for large networks, DCTCP outperforms TCP with average delay almost 45 % less as compared to TCP due to efficient use of buffer space. However, TCP can be preferred for small networks.

Incast congestion sets in early when all senders and receiver are connected to the same TOR switch as compared to the scenario when all senders are not connected to the same TOR switch as reflected in Fig. 7. Incast congestion is more serious for within rack network as compared to out of rack traffic. Figure 7 also shows that incast congestion sets in early in three-tier topology than in fat-tree topology. Similar results are obtained for outcast congestion. Hence incast and outcast concerns are serious in three-tier network topology especially for applications with flows within a TOR switch. In fat-tree topology incast and outcast congestion affects throughput more profoundly when out of rack flows increase.

## Related work

As primary focus of this research is to investigate transport protocols in data center network topologies therefore an important category of literature review comprises various DCN architectures proposed over the last decade. The design goals for these architectures vary and broadly concern performance or efficiency improvement, data latency reduction, congestion mitigation, meeting application requirements etc. Some of the notable DCN architectures include the legacy three-tier, fat-tree, DCell, BCube etc. (Al-Fares et al. 2008; Greenberg et al. 2009a; Guo et al. 2009). Greenberg et al. (2009b) proposed VL2 to improve scalability and flexibility. Guo et al. (2009) proposed BCube; a high performance server-centric architecture for DCN. Bilal et al. (2012, 2013) carried out a comparative study of three DCN architectures, one from each category; legacy, switch-based and hybrid. They selected the legacy three-tier, switch-based fat-tree and hybrid DCell architectures. They simulated these topologies using the packet level simulator NS3 (NS-3 2014) and compared throughput and average packet latencies. They used uniform random distribution and exponential random distribution for computing the communication pattern and traffic generation. Their results show that fat-tree architecture outperforms DCell and three-tier in terms of network throughput and average delay. We conclude that the legacy three-tier and fat-tree architectures are the most promising and widely deployed DCN architectures for implementation and testing of protocols and algorithms proposed for data centers.

We studied TCP challenges in data center networks and approaches to overcome these challenges. The TCP incast problem is discussed in detail by Chen et al. (2012). They state that incast congestion occurs when multiple senders linked with the same Ethernet switch transmit data to the same receiver which is a common traffic pattern in DCN. They identify that this situation occurs due to a complex interplay between datacenter applications, switches, network topology and unsuitability of TCP for datacenter networks. Another important TCP challenge is the TCP outcast problem which is identified by Prakash et al. (2012) and occurs due to taildrop queue management scheme in commodity switches. They observe that the commodity datacenter network switches are organized in multi-rooted topologies which lead to severe unfairness in bandwidth sharing which they term as the TCP outcast problem. They evaluate various solutions to this problem and also propose a new solution called equal-length routing. Wu et al. (2013) study TCP incast in detail and propose ICTCP for incast congestion control for TCP in data center networks. Their solution is based on the idea to design incast congestion control scheme on the receiver side.

Alizadeh et al. (2010) propose DCTCP which is a modified version of conventional TCP especially developed for data center networks. DCTCP uses Explicit Congestion Notification (ECN), which according to them is increasingly becoming available in modern data center switches. In DCTCP, the sources estimate the extent of congestion from the multi-bit feedback and react accordingly thus achieving high throughput. In a later paper, Alizadeh et al. (2011) extends the analysis of DCTCP from the viewpoint of stability, convergence and fairness. They provide a mathematical analysis of DCTCP based on fluid model and hybrid (continuous and discrete-time) models and corroborate their results with NS2 simulations. Das and Sivalingam (2013) propose TDCTCP which is a modified DCTCP which increases throughput without increasing delay significantly. While Alizadeh et al. perfected their scheme through mathematical analysis and simulations targeting stability, convergence and fairness comparisons with TCP and others have tried to improve DCTCP we have augmented analysis of DCTCP through simulations involving complete unabridged three-tier and fat-tree DCN topologies and multiple traffic patterns.

The traffic patterns of data center networks are analyzed and explained in detail in Benson et al. (2009, 2010), Kandula et al. (2009), Chen et al. (2011), Ersoz et al. (2007). Benson et al. (2009) present experimental analysis of end-to-end traffic patterns in data centers. The authors analyze Simple Network Management Protocol (SNMP) logs gathered at 19 data centers and observe temporal and spatial changes in link loads and losses. They examine that the links in the core are highly utilized as compared to aggregation and edge layers. Benson et al. (2010) present results of empirical study of traffic patterns of 10 data centers belonging to three different types of organizations which include university, enterprise and cloud data centers. Kandula et al. (2009) present measurement and analysis of the nature of data center traffic by gathering detailed information about traffic in data centers and congestion conditions using a cluster of 1500 server and taking measurements over 2 months. These findings have helped us in understanding data center network traffic patterns in order to generate complex and realistic traffic patterns in our simulations.

## Conclusion

This research analyzes DCTCP over three-tier and fat-tree topologies of data center network for traffic patterns spanning complete network interconnect. We realize the following goals: (a) to study whether underlying DCN topology affects congestion control schemes such as DCTCP, (b) to observe the implications when diverse traffic patterns of DCN applications are used and (c) to study incast and outcast congestion issues under above mentioned conditions. Our major findings are summarized as follows: (a) DCTCP is a promising protocol for congestion control in large data centers while TCP performs well when network size is small, (b) when network size is small, the underlying DCN topology does not affect DCTCP, however as network size increases, fat-tree gives better results than the three-tier topology, (c) DCTCP reduces delay considerably as compared to throughput improvement hence it is more suitable for applications which are delay sensitive, and (d) incast and outcast congestion are more serious for within rack traffic and in three-tier topology and DCTCP helps alleviate their impact better than TCP. This research serves as a benchmark in identifying the tight coupling between congestion mitigation schemes and the underlying DCN topologies therefore DCN topology must be an important consideration in the design of these schemes and protocols. An important future direction is to design topology specific congestion control schemes taking into account a wider range of DCN topologies and applications.

## Abbreviations

DCN: data center network
TCP: transmission control protocol
DCTCP: data center TCP
ECN: explicit congestion notification
AQM: active queue management
NS2: network simulator
GigE: gigabit ethernet
SNMP: simple network management protocol
TOR: top of rack
RTT: round trip time
ECMP: equal cost multi path
